# Disclosing the Genetic Structure of Brazil through Analysis of Male Lineages with Highly Discriminating Haplotypes

**DOI:** 10.1371/journal.pone.0040007

**Published:** 2012-07-10

**Authors:** Teresinha Palha, Leonor Gusmão, Elzemar Ribeiro-Rodrigues, João Farias Guerreiro, Ândrea Ribeiro-dos-Santos, Sidney Santos

**Affiliations:** 1 Instituto de Ciências Biológicas, Universidade Federal do Pará, Belém, Brazil; 2 IPATIMUP, Institute of Molecular Pathology and Immunology of University of Porto, Porto, Portugal; Erasmus University Medical Center, The Netherlands

## Abstract

In a large variety of genetic studies, probabilistic inferences are made based on information available in population databases. The accuracy of the estimates based on population samples are highly dependent on the number of chromosomes being analyzed as well as the correct representation of the reference population. For frequency calculations the size of a database is especially critical for haploid markers, and for countries with complex admixture histories it is important to assess possible substructure effects that can influence the coverage of the database. Aiming to establish a representative Brazilian population database for haplotypes based on 23 Y chromosome STRs, more than 2,500 Y chromosomes belonging to Brazilian, European and African populations were analyzed. No matter the differences in the colonization history of the five geopolitical regions that currently exist in Brazil, for the Y chromosome haplotypes of the 23 studied Y-STRs, a lack of genetic heterogeneity was found, together with a predominance of European male lineages in all regions of the country. Therefore, if we do not consider the diverse Native American or Afro-descendent isolates, which are spread through the country, a single Y chromosome haplotype frequency database will adequately represent the urban populations in Brazil. In comparison to the most commonly studied group of 17 Y-STRs, the 23 markers included in this work allowed a high discrimination capacity between haplotypes from non-related individuals within a population and also increased the capacity to discriminate between paternal relatives. Nevertheless, the expected haplotype mutation rate is still not enough to distinguish the Y chromosome profiles of paternally related individuals. Indeed, even for rapidly mutating Y-STRs, a very large number of markers will be necessary to differentiate male lineages from paternal relatives.

## Introduction

From the genetic point of view, Brazil is known as one of the most heterogeneous population in the world, with an important genetic contribution from three main continental groups: Europeans, Africans and Native Americans.

The first people arriving in Brazil were Europeans, coming mainly from Portugal, who arrived in 1500 to a territory that was already inhabited by the Native Americans for at least 11,000 years [1].

During the slave trade period, which officially lasted from 1538 to 1850, a huge number of African people were forced to move to Brazil. During that period, approximately 3.6 million slaves are estimated to have entered the country [2], [3]. After the abolition of slavery in Brazil in 1888, a new important migration wave took place, extending the admixed process to new European immigrants. The number of new incomers was approximately 6 million, coming from Portugal, Italy, Spain, Germany, Syria, Lebanon and Japan [4].

At the same time that people from diverse countries and continents were arriving to different regions in Brazil, important movements were taking place inside the territory, mainly due to economic interests. These internal movements gained a new impetus after the First World War, between 1914 and 1918, mainly from the northeast to the north and southeast regions of the country [5].

Consequently, the modern Brazilian population is genetically very diverse and considered to be very heterogeneous when considering the 5 main geopolitical regions of the country: (i) the northern region hosts the people with the largest Native American ancestry; (ii) northeast region has the highest African contribution; (iii) the southeast and (iv) the south are the regions where the European contribution is more important, and; (v) the central west was the last colonized region by the influx of people coming from all the other Brazilian regions, mainly from the northeast and southeast [4], [6].

Large efforts for data collection are therefore required to actually capture the genetic diversity expected in such a large and heterogeneous country. Representative population databases are very important to correctly define allele, haplotype and genotype frequency distributions, which is essential for accurate statistical inferences in i) kinship analysis or identification in criminal cases; ii) in the study of the origins and history of human populations and their genetic relationships; and iii) to study different events, like selection, that can be acting on populations [7].

Very high diversity levels can be obtained when studying a large number of Y chromosome specific loci. In most human populations, a relatively small number of 12 to 17 STRs is expected to produce a percentage of more than 90% different haplotypes in most medium-sized population samples of 100 to 500 non-related individuals (see [8]–[17] for some examples).

Although several studies have been published concerning the Y-STR variability in a large number of populations worldwide, major concerns still exist on the weight of most databases available for a wide range of applications, for example, those used to estimate haplotype frequencies for forensic investigation purposes [18]. A strong statistical evaluation of forensic evidences based on Y chromosome genetic profiles can only be obtained if it is supported by a sample that is sufficiently large to represent the diversity present in the reference population. Moreover, in admixed and/or substructured populations, the variability existing between different regions and ethnic groups should be reflected in the composition of the global database [19].

Concerning the Brazilian population, the available Y-STR haplotypic information is still poor, being represented by a relatively low number of samples for a limited group of markers. Most studies include no more than the 9 Y-STR loci from the so called “minimal haplotype” [20] or the 17 Y-STR set of the commercial kit YFiler (Applied Biosystems), which are the most widely used in forensic genetics. Still, the power of discrimination for these markers is not enough to avoid a significant proportion of shared haplotypes within the studied population samples [8]–[14].

In the present work, we analyzed more than 2,000 Y chromosomes from the five different Brazilian regions aiming to establish a representative population database of the country for haplotypes based on 23 Y chromosome STRs. In comparison to the more commonly used sets of 9 to 17 Y-STRs, we also aimed to evaluate the capacity of this extended battery of 23 markers to discriminate non-related individuals, as well as to calculate the expected probability of finding different profiles in paternal relatives due to mutation(s).

## Results and Discussion

**Figure 1:**
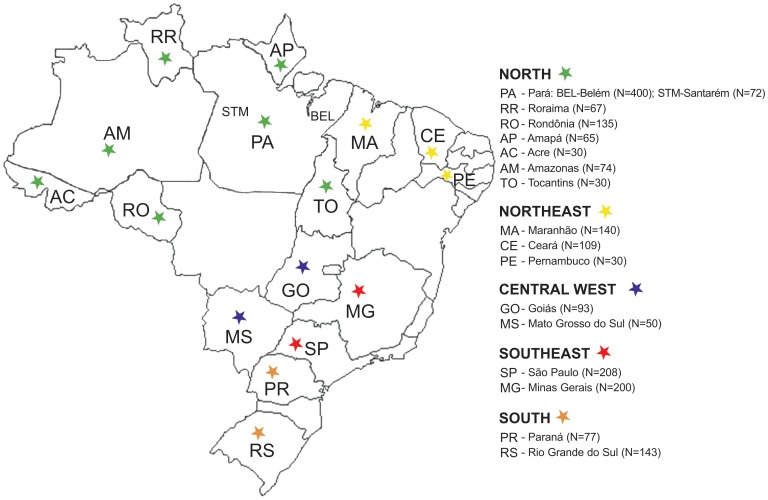
Map of Brazil indicating the geographical location of the 17 different sampling places located in 5 geopolitical regions.

A set of 23 Y chromosome STRs was investigated in a sample of admixed Brazilians from 17 different cities (Fig. 1) to evaluate the differences that could exist within and between populations from the 5 studied regions of Brazil. For this purpose, we studied three reference samples from Native Americans, Europeans and Africans because they represent the main ancestral sources of modern Brazilian populations. A list of the observed haplotypes in all studied populations from Brazil (admixed and native samples), Europe (Portugal) and Africa (Ivory Coast, Cameroon, Mozambique, Democratic Republic of the Congo, Angola) is included in Table S1.

The compiled data were used to compare the Y-STR haplotype genetic profiles of the 17 different admixed populations in Brazil grouped in 5 different geopolitical regions. Additionally, the overall diversity found in the 5 different regions in Brazil was also compared with that observed in other populations.

### Genetic Distances and Molecular Variance Analysis

Pairwise genetic distances were estimated based on the sum of squared size differences (RST) between the haplotype distributions observed for the 23 Y-STRs in the 17 Brazilian admixed population samples. No significant genetic distances were found between any pair of populations within or among the five different regions of Brazil, except in the comparison of Roraima with Amazonas and Mato Grosso do Sul (Table S2).

**Figure 2:**
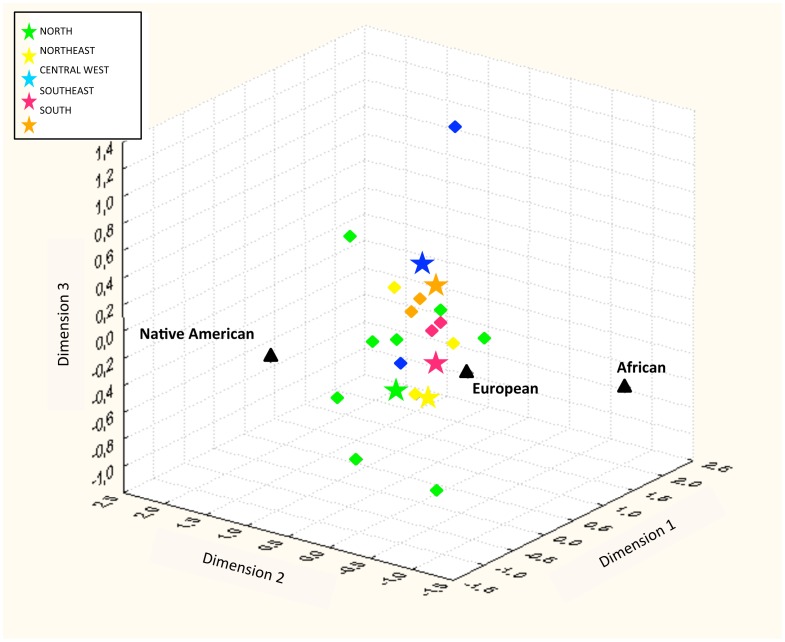
MDS plot based on the pairwise R_ST_ genetic distance matrix (Stress = 0.134).

In the MDS representation of pairwise R_ST_ genetic distances (Fig. 2), we can see that all the studied populations stand very close to the Europeans, showing a low Native American or African paternal contribution, which is repeatedly observed in admixed urban samples from different Brazilian populations studied to date (e.g., [15]–[17]).

To see if the observed Y-haplotype distribution is structured by region, we have also included in the MDS plot (Fig. 2) the samples corresponding to the 5 different Brazilian regions, obtained after pooling the data from the 17 urban samples. No patterns emerge from these analyses, and the Brazilian populations are dispersed on the plot without any kind of grouping around the region with which they belong. There are, nevertheless, two populations that stand apart from the main group, Roraima and Mato Grosso do Sul. These populations present significantly high values of genetic distances to all of the three reference groups from Europe, Africa and Native America and, therefore, the observed results cannot be explained by a higher proportion of African or Native American chromosomes but instead it seems to be the result of drift. This slight differentiation of Roraima is supported by the low non-differentiation *p*-values, which in two cases presented statistical significance (Table S2).

Nevertheless, no conclusions can be drawn about the sample from Mato Grosso do Sul if we take into account that in this case the high R_ST_ values are associated with non-differentiation *p* values clearly above the significance level, meaning that although drift exists, it can be a consequence of a low sample size. Finally, when grouping the 17 populations in the five different Brazilian regions, AMOVA showed that almost all genetic variation is within populations (fixation indexes: F_SC_ = 0.00055, F_ST_ = 0.00002 and F_CT_ = −0.00053), with no significant variation within or between groups (significant test after 10100 permutations, *p≥*0.30).

In summary, for the 23 STRs included in the present work, no significant differences could be detected concerning haplotype distributions within or between the northern, northeastern, central western, southeastern and southern regions. Therefore, subsequent analyses were made just taking into account these 5 regions or the entire Brazilian population.

### Analysis of Genetic Diversity

To evaluate the utility of increasing the number of STRs in population and forensic genetics, we compared the results obtained when using four different groups of markers: (i) the 9-set belonging to the minimal haplotype (MH) described by Kayser et al. [20]; (ii) the 17-set loci included in the YFiler kit; (iii) a 14-set comprising the 8 YFiler loci not included in the MH plus 6 new selected loci; (iv) and the total set of 23 markers included in the present work.

The diversity indices, calculated using the four different Y-STR sets in the five Brazilian regions, are presented in Table 1, which also includes the results obtained for the three reference population samples.

**Table 1:** Single locus and haplotype genetic diversities calculated for the 5 different regions as well as in the total sample from Brazil.

	Brazil								
Loci	North	Northeast	Central West	Southeast	South	Brazil	Africa	Native America	Portugal
DYS392	0,628	0,620	0,608	0,593	0,603	0,616	0,201	0,332	0,585
DYS391	0,574	0,541	0,572	0,564	0,576	0,568	0,348	0,381	0,594
DYS390	0,657	0,724	0,660	0,666	0,681	0,673	0,444	0,679	0,689
DYS393	0,507	0,502	0,532	0,438	0,486	0,491	0,632	0,364	0,507
DYS389 I	0,625	0,580	0,544	0,489	0,522	0,576	0,460	0,424	0,536
DYS389 II	0,687	0,722	0,727	0,695	0,673	0,694	0,703	0,702	0,665
DYS19	0,649	0,645	0,630	0,643	0,658	0,647	0,643	0,322	0,679
DYS385	0,914	0,919	0,902	0,886	0,860	0,901	0,947	0,869	0,898
DYS458	0,791	0,791	0,799	0,791	0,797	0,792	0,727	0,644	0,749
DYS439	0,681	0,677	0,705	0,660	0,678	0,678	0,656	0,537	0,548
Y-GATA H4	0,578	0,632	0,645	0,596	0,633	0,606	0,579	0,637	0,564
DYS456	0,689	0,711	0,666	0,682	0,664	0,685	0,563	0,666	0,708
DYS437	0,585	0,625	0,597	0,588	0,623	0,599	0,202	0,406	0,607
DYS635	0,761	0,727	0,788	0,778	0,733	0,763	0,611	0,468	0,718
DYS448	0,673	0,699	0,693	0,699	0,691	0,687	0,578	0,577	0,700
DYS438	0,696	0,703	0,666	0,700	0,673	0,693	0,448	0,255	0,678
DYS576	0,821	0,795	0,793	0,778	0,774	0,800	0,802	0,704	0,812
DYS447	0,724	0,743	0,700	0,692	0,728	0,719	0,729	0,635	0,814
DYS460	0,552	0,600	0,562	0,577	0,593	0,573	0,529	0,333	0,551
Y-GATA A10	0,652	0,682	0,709	0,660	0,669	0,666	0,688	0,645	0,584
DYS449	0,833	0,848	0,832	0,834	0,815	0,833	0,859	0,826	0,818
DYS570	0,776	0,791	0,733	0,777	0,795	0,779	0,821	0,713	0,776
**Mean values:**									
09/set	0.655	0.656	0.647	0.622	0.632	0.646	0.547	0.509	0.644
14/set	0.701	0.716	0.706	0.701	0.705	0.705	0.628	0.575	0.688
17/set	0.668	0.676	0.671	0.654	0.659	0.667	0.546	0.516	0.652
23/set	0.684	0.694	0.685	0.672	0.678	0.684	0.599	0.551	0.672
**No. of samples**	874	279	143	408	320	2024	148	310	50
09/set									
No. of haplot.:	663	225	122	287	250	1270	–	–	–
No. unique haplot.:	569	198	109	240	230	1010	–	–	–
Haplot. diversity:	0.9969	0.9937	0.9881	0.9908	0.9910	0.9959	–	–	–
14/set									
No. of haplot.:	871	277	143	408	320	2002	–	–	–
No. unique haplot.:	869	275	143	408	320	1977	–	–	–
Haplot. diversity:	0.9988	0.9964	0.9930	0.9975	0.9969	0.9995	–	–	–
17/set									
No. of haplot.:	862	274	143	401	318	1956	–	–	–
No. unique haplot.:	851	269	143	397	316	1899	–	–	–
Haplot. diversity:	0.9987	0.9963	0.9930	0.9974	0.9968	0.9994	–	–	–
23/set									
No. of haplot.:	874	279	143	408	320	2017	–	–	–
No. unique haplot.:	874	279	143	408	320	2010	–	–	–
Haplot. diversity:	0.9988	0.9965	0.9930	0.9975	0.9969	0.9995	–	–	–

When comparing single locus diversities, similarly high values were obtained in all loci in the 5 different Brazilian regions. Nevertheless, on average, the northeastern region presents slightly higher diversity values than those observed in the remaining regions for any of the 4 selected Y-STR sets. Genetic diversities above 0.50 have been observed in all Y-STRs in north, northeast and central west. The south and southeast regions have slightly lower single locus diversities, and although not significantly different, the lowest average values calculated for the 4 Y-STR sets were always detected in the southeastern region.

In the global sample from Brazil, single locus diversities were consistently higher than in the three reference samples, which can be somewhat explained by the admixture of very heterogeneous male contributions. As observed in previous studies for lineage markers [21] as well as for markers in the autosomes [22], the Native Americans have the lowest genetic diversity. The lower levels of polymorphisms present in Africans in comparison to Europeans is most likely explained by the selection criteria of the analyzed markers that were initially based on diversity levels found in Europeans.

At the haplotype level, the same results were obtained, with northeastern Brazil showing the highest diversity and southeastern the lowest.

When analyzing the discrimination levels produced by the 5 different sets of Y-STRs, for the 9-set 50% of the haplotypes were found in more than one Y chromosome in our Brazilian sample, and the number of different haplotypes corresponds to 62% of the total sample. As expected, these values increase when using larger sets, but are higher when considering the 14-set (including the new selected markers) than the 17-set of markers in the YFiler kit (see haplotype sharing and diversities in Table 1).

For the group of 23 Y-STRs included in this study, out of 2,024 Y chromosomes that were typed from the Brazilian admixed populations, a total of 2,010 were found only once, with just seven chromosomes being shared between different samples, namely Pará (Belém) and Minas Gerais; Amapá and Maranhão; Rondônia and Rio Grande do Sul; Rondônia and Ceará; Amazonas and Maranhão; Maranhão and São Paulo; and São Paulo and Rio Grande do Sul.

In summary, the 6 additional STRs allowed an increased discrimination capacity of Yfiler, the commercial kit most widely used in Y-STR typing, by decreasing the percentage of shared haplotypes between individuals from 6.2% to 0.3% and the matching probability from 1 in 1,700 to 1 in 2,000.

### Ability to Distinguish Paternal Relatives

The study of Y-STRs can be very useful in some particular applications, namely in cases of genealogy or kinship investigation, or in forensic identification, especially when female/male mixtures need to be analyzed [23]. In any of these situations, besides knowing haplotype frequencies, it is also very important to estimate the probability of finding different haplotypes in close relatives due to mutation in one or more loci.

Usually, the first concern when selecting Y-STRs is to find a group of markers that allow obtaining highly informative haplotypes in the population used as reference, which is associated not only with the number of Y-STRs under analysis but also with each locus diversity as well as with linkage disequilibrium between alleles at the different selected loci. Nevertheless, no matter the levels of polymorphism, because Y chromosome markers are transmitted without recombination, males that are paternal relatives will share the same Y-haplotype unless a mutation event takes place. This can be a strong limitation in identification or paternity cases when multiple suspects or presumed fathers belong to the same patrilineage. In these situations, distinction of two paternal relatives will be directly related with the mutation rate of the full haplotype and the number of meioses that separates both individuals.

Therefore, we calculated the expected probability of finding different profiles in paternal relatives due to mutation(s). We applied the same formula as Gusmão et al. [24] but used locus specific estimates to calculate the mutation rate of the complete haplotype. The mutation rate of a full haplotype was calculated as the probability of at least one copy error occurring in the meiotic transmission of its *n* loci. Therefore, the probability of distinguish two paternal related Y chromosome haplotypes is:
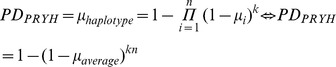
where *n* is the number of loci in the haplotype, *k* is the number of meioses separating two haplotypes, µ*_i_* is the mutation rate of the i-th locus and µ_average_ is the average mutation rate calculated for all loci included in the haplotypes.

Table 2 presents the results obtained for haplotypes based on the four previously defined sets of 9, 14, 17 and 23 Y-STRs. If two individuals are separated by a single meiosis (and they are father and son), the probability of having two different haplotypes will vary from 2% to 9%, for the 9 and 23 sets, respectively. The addition of the 6 extra markers to the YFiler set doubles the probability of finding different haplotype profiles between close relatives.

**Table 2:** Probability of finding at least one mutation between two Y-STR haplotypes one to four generations apart.

Y-STR	Mutation rate*	One Generation Prob.of at least one mutation		Two Generations Prob.of at least one mutation		Three Generations Prob.of at least one mutation		Four Generations Prob.of at least one mutation	
		%	1 in:	%	1 in:	%	1 in:	%	1 in:
DYS19	0,002299	0.23	435	0.46	218	0.69	145	0.92	109
DYS389I	0,002523	0.25	396	0.50	198	0.75	132	1.01	99
DYS389II	0,003644	0.36	274	0.73	137	1.09	92	1.45	69
DYS390	0,002102	0.21	476	0.42	238	0.63	159	0.84	119
DYS391	0,002599	0.26	385	0.52	193	0.78	129	1.04	97
DYS392	0,000412	0.04	2425	0.08	1213	0.12	809	0.16	607
DYS393	0,001045	0.10	957	0.21	479	0.31	319	0.42	240
DYS385a	0,002080	0.21	481	0.42	241	0.62	161	0.83	121
DYS385b	0,004140	0.41	242	0.83	121	1.24	81	1.65	61
DYS458	0,006444	0.64	155	1.28	78	1.92	52	2.55	39
DYS439	0,005214	0.52	192	1.04	96	1.56	64	2.07	48
GATA H4	0,002434	0.24	411	0.49	206	0.73	137	0.97	103
DYS456	0,004243	0.42	236	0.85	118	1.27	79	1.69	59
DYS437	0,001226	0.12	816	0.25	408	0.37	272	0.49	204
GATA C4/DYS635	0,003467	0.35	288	0.69	144	1.04	96	1.38	72
DYS448	0,001571	0.16	637	0.31	319	0.47	213	0.63	160
DYS438	0,000306	0.03	3269	0.06	1635	0.09	1090	0.12	818
DYS576	0,014300	1.43	70	2.84	35	4.23	24	5.60	18
DYS447	0,002120	0.21	472	0.42	236	0.63	158	0.85	118
DYS460	0,006220	0.62	161	1.24	81	1.85	54	2.46	41
GATA A10	0,003320	0.33	301	0.66	151	0.99	101	1.32	76
DYS449	0,012200	1.22	82	2.43	41	3.62	28	4.79	21
DYS570	0,012400	1.24	81	2.46	41	3.67	27	4.87	21
09-Set	0.0023	2.07	48	4.09	24	6.07	16	8.01	12
14-Set	0.0054	7.29	14	14.04	7	20.31	5	26.12	4
17-Set	0.0027	4.48	22	8.75	11	12.84	8	16.74	6
23/set	0.0042	9.20	11	17.55	6	25.14	4	32.03	3
69-Set	0.01	50.016							
34-Set	0.02	49.686							
916-Set	0.01	99.990							
458-Set	0.02	99.990							

* Single locus mutation rates are according to YHRD (www.yhrd.org) and Ballantyne et al. [35]; haplotype mutation rates for the different sets have been calculated as the average mutation of the corresponding.

Therefore, even for close relatives, a larger set of markers will be definitely useful to increase the chance of Y-haplotype exclusion, which is strongly related to the mutation rate of the included markers. Still, in most cases, even for individuals separated by four meiosis (as is the case for two cousins, for example), no differences will be found in almost 60% of the cases and results based on Y-STRs will present a limitation at the individual identification level when paternal relatives are in question.

This is a limitation that is far from being solved with a restricted set of markers, even with exceptionally high mutation rates, as previously shown by Ballantyne et al. [25]. In this study, based on a set of 14 rapidly mutating Y-STR loci (corresponding to 13 markers, one with two loci) in pairs of relatives, the genotyping results showed that in more than 50% of the studied cases there was no difference between father and son. Indeed, if we consider that there is no association between different mutation events, we will need to type a total number of 34 Y-STRs with an average mutation rate of 2% to obtain a 50% chance of finding a difference between father and son. A 99.99% probability of finding differences between any male relatives will require very large sets of quickly mutating markers. To reach this probability, for STRs with an average mutation rate of 0.02 (like those selected by Ballantyne et al. [25]), it is necessary to type a group of at least 115 markers for haplotypes separated by four meiotic transmission, a number that increases to 458 in father-son pairs (Table 2).

### Conclusions

In all genetic studies that are based on haploid segments of the genome, as is the case for mtDNA and the Y chromosome it is very important to have reliable estimates of haplotype frequencies, for which large databases with good coverage are critical [8]. The high susceptibility of lineage markers to genetic drift is responsible for larger genetic distances between populations and population substructure, especially in admixed populations.

Thus far, there is no agreement on the best approach to calculate accurate frequencies for rare Y chromosome haplotypes [26]–[28]. However, no matter the estimation approach we choose, if no substructure exists, pooling local population samples will produce a larger and therefore better represented Y-haplotype database.

Aiming to produce a large representative database of Brazil for highly discriminating Y chromosome-specific haplotypes, we have sampled more than 2,000 Brazilian chromosomes. Despite the different colonization history of each region in Brazil, it was possible to see that all populations included in the present work have very similar haplotype profiles concerning the 23 studied Y-STRs. This apparent lack of genetic heterogeneity is in accordance with previous reports that show a predominance of European male lineages in all regions of the country, in contrast with the much more admixed pattern of the maternal ancestry. There is an absence of a strong regional substructure in Brazil, which was also recently reported by Pena et al. [29] based on autosomal ancestry informative markers. Therefore, if we do not consider the diverse Native American or Afro-descendent isolates, which are spread throughout the country, a single Y chromosome haplotype frequency database will adequately represent the urban populations in Brazil.

The analyses of STRs in a large number of Y chromosomes belonging to Brazilian populations supports the need of using an extended battery of markers to increase haplotype diversity, allowing for a higher discrimination capacity in criminal investigation [30]. In accordance with the previously sustained by Ballantyne et al. [25], the selection of rapidly mutating STR markers will allow an increased capacity to discriminate paternal relatives. Nevertheless, a very large number of STRs will be necessary to allow the use of Y chromosome information for forensic interpretation at an individual level in cases of haplotype match.

## Materials and Methods

### Population Samples

The present work follows the ethical principles stated in the Helsinki Declaration (2000) of the World Medical Association, and approved by the institutional review board of the Laboratório de Genética Humana e Médica, Instituto de Ciências Biológicas, Universidade Federeal do Pará. Samples involved in the study are long-lasting anonymized DNA extracts previously obtained with informed written consent from healthy individuals for research purposes. Blood samples and buccal swabs were collected from 2,024 non-related males from different Brazilian states representing five geopolitical regions (see Fig. 1 for sample locations and sizes). DNA was extracted as described previously [31] and quantified using the NanoDrop 100 (NanoDrop Technologies, USA).

### Y-STR Typing Conditions

Samples were typed for 23 STRs in two PCR multiplex reactions: Multiplex I (DYS393, DYS19, DYS390, DYS389 I, DYS389 II, DYS392, DYS391, DYS385 I/II) and Multiplex II (DYS458, DYS439, Y-GATA H4, DYS576, DYS447, DYS460, DYS456, Y-GATA A10, DYS437, DYS449, DYS570, Y-GATA C4, DYS448 e DYS438). Primer sequences, PCR conditions, separation and detection and alleles assignment and nomenclature were previously described by Palha et al. [32], [33].

### Data Analysis

Haplotype diversity was calculated using the formula:
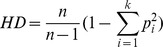
where *n* is the number of gene copies in the sample, *k* is the number of haplotypes, and *p_i_* is the sample frequency of the *i*-th haplotype.

The total number of observed and unique haplotypes, single locus diversity, AMOVA and pairwise genetic distances (RST) were calculated with the software Arlequin 3.5.1.2 [34] and visualized as a two-dimensional graphic with the Multi Dimensional Scaling (MDS) method implemented in the software STATISTICA 7.0 (StatSoft Inc. 2004).

## Supporting Information

Table S1 **List of Y-chromosome STR haplotypes found in 17 different admixed populations in Brazil, grouped in 5 different geopolitical regions, and in Native Americans, Europeans and Africans.**
(XLS)

Table S2 **Matrix showing the pairwise R_ST_ values between Brazilian populations obtained for the haplotypes defined by the whole 23-markers set (F_ST_ values below diagonal) and the corresponding differentiation** ***p*** **values (above diagonal).**
(DOC)
