# Supplementary material for: Disclosing the Genetic Structure of Brazil through Analysis of Male Lineages with Highly Discriminating Haplotypes
Source: PLoS One. 2012 Jul 10;7(7):e40007. doi: 10.1371/journal.pone.0040007 (PMC3393733; doi:10.1371/journal.pone.0040007)
Supplement: Table S2 — Matrix showing the pairwise RST values between Brazilian populations obtained for the haplotypes defined by the whole 23-markers set (FST values below diagonal) and the corresponding differentiation p values (above diagonal). (DOC) [file pone.0040007.s002.doc]

**Table S2** Matrix showing the pairwise R_ST_ values between Brazilian populations obtained for the haplotypes defined by the whole 23-markers set (F_ST_ values below diagonal) and the corresponding differentiation *p* values (above diagonal)

| **BRAZIL** | | | | | | | | | | | | | | | | | |
| --- | --- | --- | --- | --- | --- | --- | --- | --- | --- | --- | --- | --- | --- | --- | --- | --- | --- |
| **REGIONS/**  **CITIES** | **North** | | | | | | | | **Northeast** | | | **Central West** | | **Southeast** | | **South** | |
|  | BEL | AC | AP | AM | RO | RR | STM | TO | CE | MA | PE | GO | MS | MG | SP | PR | RS |
| BEL | * | 0.18325 | 0.11217 | 0.13474 | 0.45698 | 0.16177 | 0.11811 | 0.61776 | 0.26324 | 0.46035 | 0.41699 | 0.37442 | 0.07247 | 0.52084 | 0.70894 | 0.49282 | 0.58341 |
| AC | -0.00431 | * | 0.53906 | 0.60509 | 0.53292 | 0.11246 | 0.11791 | 0.11613 | 0.40709 | 0.41125 | 0.75775 | 0.77547 | 0.15553 | 0.48728 | 0.45114 | 0.47698 | 0.34403 |
| AP | 0.00441 | -0.00332 | * | 0.10197 | 0.19850 | 0.02346 | 0.07781 | 0.06692 | 0.40600 | 0.11336 | 0.23829 | 0.75547 | 0.02950 | 0.16988 | 0.25958 | 0.37749 | 0.16286 |
| AM | 0.00544 | -0.00407 | 0.00842 | * | 0.35907 | 0.00040 | 0.23532 | 0.07138 | 0.50886 | 0.14731 | 0.12920 | 0.81368 | 0.04346 | 0.24344 | 0.35907 | 0.28908 | 0.26888 |
| RO | -0.00235 | -0.00906 | 0.00110 | 0.00063 | * | 0.28175 | 0.32106 | 0.99545 | 0.76190 | 0.62984 | 0.83071 | 0.56925 | 0.05158 | 0.66766 | 0.61925 | 0.87793 | 0.56440 |
| RR | 0.00379 | 0.01285 | 0.01896 | 0.03706 | 0.00048 | * | 0.00030 | 0.00366 | 0.37204 | 0.10524 | 0.04188 | 0.43867 | 0.06504 | 0.26443 | 0.37066 | 0.39570 | 0.32789 |
| STM | 0.00574 | 0.01300 | 0.01072 | 0.00339 | 0.00065 | 0.04316 | * | 0.28284 | 0.50411 | 0.15464 | 0.05059 | 0.65825 | 0.04217 | 0.19721 | 0.34274 | 0.29621 | 0.22711 |
| TO | -0.00829 | 0.01748 | 0.01808 | 0.01588 | -0.01512 | 0.04413 | 0.00377 | * | 0.99475 | 0.95971 | 0.23889 | 0.97248 | 0.28542 | 0.90288 | 0.88051 | 0.97149 | 0.60687 |
| CE | -0.00051 | -0.01200 | -0.00089 | -0.00141 | -0.00644 | 0.00087 | -0.00169 | -0.01805 | * | 0.66825 | 0.70330 | 0.51629 | 0.03277 | 0.49193 | 0.47530 | 0.80784 | 0.30581 |
| MA | -0.00221 | -0.00764 | 0.00288 | 0.00345 | -0.00617 | 0.00395 | 0.00251 | -0.01390 | -0.00610 | * | 0.69765 | 0.57351 | 0.06425 | 0.65162 | 0.60073 | 0.91100 | 0.45144 |
| PE | -0.00677 | -0.01072 | 0.00584 | 0.01056 | -0.01207 | 0.02173 | 0.01990 | 0.00786 | -0.01478 | -0.01113 | * | 0.94832 | 0.24334 | 0.80487 | 0.75616 | 0.75824 | 0.54985 |
| GO | -0.00207 | -0.01435 | -0.00410 | -0.00325 | -0.00617 | 0.00013 | -0.00207 | -0.01737 | -0.00810 | -0.00617 | -0.01696 | * | 0.01901 | 0.58539 | 0.36986 | 0.50975 | 0.27770 |
| MS | 0.02032 | 0.02330 | 0.05092 | 0.05355 | 0.02513 | 0.04769 | 0.05279 | 0.01608 | 0.03720 | 0.02566 | 0.01897 | 0.03218 | * | 0.05881 | 0.09563 | 0.26690 | 0.06485 |
| MG | -0.00211 | -0.00792 | 0.00242 | 0.00242 | -0.00527 | 0.00099 | 0.00296 | -0.01174 | -0.00428 | -0.00471 | -0.01095 | -0.00533 | 0.02908 | * | 0.79418 | 0.69072 | 0.65875 |
| SP | -0.00286 | -0.00729 | 0.00293 | 0.00292 | -0.00466 | 0.00194 | 0.00298 | -0.01143 | -0.00302 | -0.00443 | -0.01001 | -0.00393 | 0.01416 | -0.00422 | * | 0.86744 | 0.78507 |
| PR | -0.00536 | -0.01114 | 0.00092 | 0.00137 | -0.00976 | 0.00058 | 0.00121 | -0.01727 | -0.00991 | -0.00968 | -0.01448 | -0.00955 | 0.01726 | -0.00825 | -0.00756 | * | 0.62519 |
| RS | -0.00213 | -0.00365 | 0.00686 | 0.00654 | -0.00317 | 0.00414 | 0.00704 | -0.00770 | -0.00028 | -0.00236 | -0.00641 | -0.00091 | 0.01318 | -0.00287 | -0.00393 | -0.00584 | * |
